# Copper-Based Diamond-like Thermoelectric Compounds: Looking Back and Stepping Forward

**DOI:** 10.3390/ma16093512

**Published:** 2023-05-03

**Authors:** Wenying Wang, Lin Bo, Junliang Zhu, Degang Zhao

**Affiliations:** School of Materials Science and Engineering, University of Jinan, Jinan 250022, China

**Keywords:** thermoelectric, copper-based diamond-like compounds, *zT*, lattice conductivity, device

## Abstract

The research on thermoelectric (TE) materials has a long history. Holding the advantages of high elemental abundance, lead-free and easily tunable transport properties, copper-based diamond-like (CBDL) thermoelectric compounds have attracted extensive attention from the thermoelectric community. The CBDL compounds contain a large number of representative candidates for thermoelectric applications, such as CuInGa_2_, Cu_2_GeSe_3_, Cu_3_SbSe_4_, Cu_12_SbSe_13_, etc. In this study, the structure characteristics and TE performances of typical CBDLs were briefly summarized. Several common synthesis technologies and effective strategies to improve the thermoelectric performances of CBDL compounds were introduced. In addition, the latest developments in thermoelectric devices based on CBDL compounds were discussed. Further developments and prospects for exploring high-performance copper-based diamond-like thermoelectric materials and devices were also presented at the end.

## 1. Introduction

The attractive capability of thermoelectric (TE) materials in actualizing the conversion between temperature gradient and electrical power makes them strong candidates for waste-heat recovery as well as solid-state refrigeration [1,2,3]. The practical and widespread application of TE technology strongly relies on the development of high-performance TE materials, where the TE performance of materials is evaluated by a dimensionless figure of merit, *zT* = *α*^2^*σT*/*κ*. The TE parameters *α* and *σ* are the Seebeck coefficient and electrical conductivity which, respectively, constitute the power factor, *PF* = *α*^2^*σ*, used to evaluate electrical conductivity characteristics. Parameter *T* is the Kelvin thermodynamic temperature, while *κ* refers to the total thermal conductivity, which is composed of two major contributions from the charge carriers (*κ*_E_) and the lattice (*κ*_L_), respectively. From a computational perspective, the most ideal high-performance TE material should have a large *α*, high *σ* as well as a low *κ* value. What cannot be avoided is the strong coupling between thermoelectric parameters regarding carrier concentration, such as when a high *σ* means low *α* and a high *κ*_E_, limiting the improvement of *zT* [4,5,6]. In order to achieve high *zT* in traditional or emerging TE materials, various methods and approaches have been adopted to reduce the correlation between thermal and electrical properties [7,8,9], including defect engineering, size effects, alloying effect and high-entropy engineering, etc. In addition to achieving high performance, the exploration of alternative materials consists of earth-abundant and eco-friendly components to meet the sake of clean and environmental protection is also considered as one of the most popular approaches in TE field [10,11,12,13]. In recent years, diverse bulk TE materials have been widely researched, including liquid-like Cu_2_(S, Se, Te), silver-based chalcogenides, Sn(Te, S, Se), half-heuslers, etc. [14,15,16].

**Figure 1:**
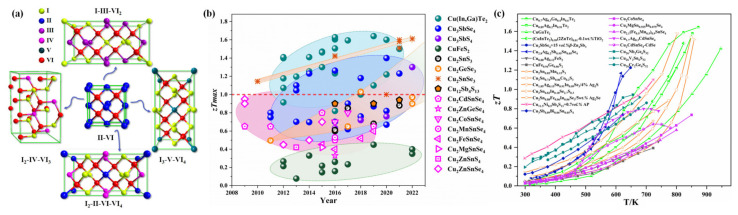
(**a**) Various crystal structures of CBDL thermoelectric compounds; timeline of *zTs* (**b**) and the temperature dependence of *zTs* (**c**) for selected copper-based diamond-like thermoelectric compounds, data culled from Cu(In, Ga)Te_2_ [23,26,29,30,31,32,33,34,35,36,37,38,39,40,41], Cu_3_SbSe_4_ [27,42,43,44,45,46,47,48,49], Cu_3_SbS_4_ [28,50,51], CuFeS_2_ [52,53,54,55], Cu_2_SnS_3_ [56,57,58], Cu_2_GeSe_3_ [59,60,61], Cu_2_SnSe_3_ [62,63,64,65,66,67], Cu_12_SbSe_13_ [68,69], Cu_2_CdSnSe_4_ [70,71,72,73,74], Cu_2_ZnGeSe_4_ [75], Cu_2_CoSnSe_4_ [76], Cu_2_MnSnSe_4_ [76], Cu_2_FeSnSe_4_ [76,77,78], Cu_2_MgSnSe_4_ [79], Cu_2_ZnSnS_4_ [33], Cu_2_ZnSnSe_4_ [33].

As an environmentally friendly and promising TE material without precious elements, the performance advantages of copper-based diamond-like TE compounds lie in their high Seebeck coefficient and low thermal conductivity [17,18,19,20,21]. Typical compounds include: Cu_3_SbSe_4_, with a high *zT* of 0.89 at 650 K [19]; Cu_2_SnSe_3_, with *α* of ~250 μV·K^−1^ in the temperature range of 300–700 K [22]; and CuInTe_2_, with a *κ_L_* value as low as 0.3 W·m^−1^·K^−1^ [23], etc. Copper-based diamond-like TE compounds are a type of material that conforms to the concept of “phonon-glass electron-crystal” (PGEC) [17] materials, and their crystal structures are usually composed of two sublattices [23,24,25], in which one sublattice constitutes a conductive network, while the other acts as a thermal barrier and is sometimes also known as a charge reservoir. In 2011, Skoug et al. [24] summarized the significance of lone-pair electrons in the Cu-Sb-Se diamond-like system and demonstrated that the low intrinsic *κ_L_* in compounds came from the interaction of lone-pair electrons with neighboring atoms. Moreover, Skoug et al. [25] also confirmed that the dominant Cu-Se network controlled the electric transport while the Sn orbitals only compensated the system for electrons. Several diamond-like crystal structures evolved from thecubic zincblende structure are shown in Figure 1a. Simultaneously, a series of advanced CBDL compounds have been discovered since 2009, most of which have presented outstanding TE properties. The timeline of maximum *zTs* and the temperature dependence of *zTs* for selected CBDL compounds are shown in Figure 1b,c. Taking the typical diamond-like compounds of Cu(In, Ga)Te_2_, Cu_3_SbSe_4_, and Cu_2_SnSe_3_ as examples, long-term efforts have shown that they all apparently have superior TE transport properties with high *zT*s that exceed one. For instance, Liu et al. [23] devised a pseudocubic crystal structure in CuInTe_2_ compounds; thus the highest *zT* of 1.24 was obtained in Ag-doped CuInTe_2_ compounds. A peak *zT* of 1.14 was attained in a Cu_2_Sn_0.90_In_0.10_Se_3_ compound at 850 K by replacing Sn sites with In. It is also worth noting that a high average *zT* (*zT_ave_*) value is desirable for overall TE conversion efficiency. For instance, a high *zT_max_* of 1.67 at 873 K, and a *zT_ave_* of 0.73, were realized in Cu_0.7_Ag_0.3_Ga_0.4_In_0.6_Te_2_ [26]. In the latest research of Zhou’s group [27,28], record-high *zT_ave_* values of 0.73 and 0.77 were achieved in Cu_3_SbSe_4_–based and Cu_3_SbS_4_–based materials, respectively, which were also comparable to other state-of-the-art TE compounds. Hence one can see that CBDL compounds are expected to become environmentally friendly candidates for TE applications and to achieve excellent performances.

In this review, the structural origins, and the decoupled transport properties of CBDL thermoelectric compounds, were summarized. The latest advances in different types of CBDL compounds were discussed. Then, several common synthetic methods of CBDL compounds were briefly introduce, typical strategies for optimizing the TE properties of CBDL compounds were described in detail, as well as recent updates on CBDL-based TE devices. Finally, the future development of CBDL thermoelectric compounds was evaluated.

## 2. Copper-Based Diamond-like Thermoelectric Compounds

CBDL compounds contain a large number of family members, which include ternary I–III–VI_2_ chalcopyrites, I_3_–V–VI_4_ stannites, I_2_–IV–VI_3_ stannites, quaternary I_2_–II–IV–VI_4_ compounds, and even large-cell Cu_10_B_2_C_4_D_13_ tetrahedrites and Cu_26_P_2_Q_6_S_32_ colusites. The TE properties of selected typical CBDL compounds including *zT_ave_*, *zT_max_*, *α^2^σ*, *κ_L_*, and carrier concentration (*n*) at room temperature are displayed in Table 1. 

Among CBDL compounds, CuGaTe_2_ and CuInTe_2_ are typical Cu–III–VI_2_ (III = In, Ga; VI = Se, S, Te) chalcopyrites structural compounds which have exhibited excellent thermoelectric properties at higher temperatures. In 2012, Plirdpring et al. [29] achieved a record *zT* of 1.4 in CuGaTe_2_ compound at 950 K, which indicated that it was a potential material in the field of TE applications. Comparatively, it was found that CuInTe_2_ possessed a high *zT* of 1.18 at 850 K [20]. A large number of studies were conducted to optimize the TE transport behaviors of chalcopyrite-based materials in the following years. Through defect engineering, Pei’s team obtained a maximum *zT* of 1.0 at 750 K in the Ag-doped CuGaTe_2_ compound [40] and identified that vacancy scattering was an active approach to improve TE transport behaviors [80]. Zhang et al. [26] synthesized a quinary alloy compound Cu_0.7_Ag_0.3_Ga_0.4_In_0.6_Te_2_ with a complex nanosized strain domain structure, which presented excellent TE properties with a peak *zT* of 1.64 at 873 K and an average *zT* (*zT_ave_*) of 0.73. Through compositing TiO_2_ nanofibers, Yang et al. [36] achieved a maximum *zT* of 1.47 at 823 K in a CuInTe_2_–based TE compound. Moreover, Chen et al. [23] obtained a maximum *zT* of 1.24 in the Cu_0.75_Ag_0.2_InTe_2_ compound. The above shows that Cu(In, Ga)Te_2_ diamond-like TE materials have a higher *zTs*, comparable to other advanced thermoelectric materials such as PbTe [81,82,83,84] and SnTe [85,86,87]. In addition, a natural chalcopyrite mineral, CuFeS_2_ [52,53,54,55], was also recognized as an advanced CBDL thermoelectric material. It is noteworthy that the CuFeS_2_ compound is a rare typical *n-*type TE compound among CBDL thermoelectric materials [88,89].

Cu_3_–V–VI_4_ (V = Sb, P, As; VI = Se, S, Te) compounds with a tetragonal diamond-like crystal structure can be approximately regarded as the superposition of four equivalent zincblendes, wherein Cu_3_SbSe_4_ is considered as a promising TE candidate owing to its narrow band gap of ~0.3 eV [19,27,47,90]. For improving the TE performance of Cu_3_SbSe_4_–based materials, Li et al. [45] coordinately regulated electrical and thermal transport behaviors through the incorporation of Sn-doping and AgSb_0.98_Ge_0.02_Se_2_ inclusion, and the highest *zT* of 1.23 was eventually achieved at 675 K. Bo et al. [90] successfully applied the concept of configuration entropy to optimize the TE performance of Cu_3_SbSe_4_, and the *zT* increased by about four times, compared to the initial phase, with the increase of entropy. In their latest report, Zhou’s group [27] attained a superior average power factor (*PF_ave_*) of 19 µW·cm^−1^·K^−2^ in 300–723 K by using a small amount of foreign Al atoms as “stabilizers” to supply the high hole concentration, with almost no effect on carrier mobility. Consequently, combined with the reduced *κ*, a record-high *zT* of 1.4 and a *zT_ave_* of 0.72 were obtained within the Cu_3_SbSe_4_–based compounds. A new unconventional doping process that can coordinate the TE properties of materials was also presented. Apart from Cu_3_SbSe_4_, Cu_3_SbS_4_ is also a promising Cu_3_–V–VI_4_–type of TE material [28,50,51], and it has been demonstrated that its *PF_ave_* can reach up to 16.1 µW·cm^−1^·K^−2^ and the *zT_ave_* up to 0.77 between 400 and 773 K via its optimization [28].

**Figure 2:**
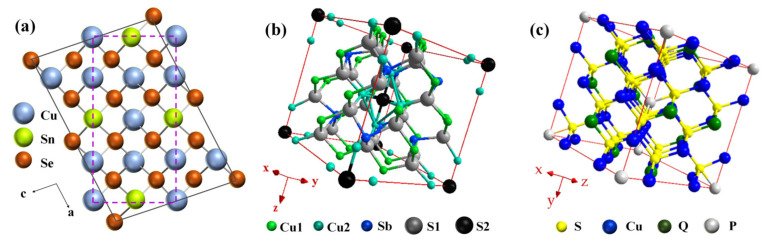
Crystal structure of: (**a**) Cu_5_Sn_2_Se_7_ (reprinted with permission from ref. [91], Copyright 2014 American Chemical Society); (**b**) Cu_12_SbS_13_ (reprinted with permission from ref. [110], copyright 2015 American Chemical Society); and (**c**) Cu_26_P_2_Q_6_S_32_ (reprinted with permission from ref. [106], copyright 2018 American Chemical Society).

Different from Cu–III–VI_2_ and Cu_3_–V–VI_4_ compounds, ternary Cu_2_–IV–VI_3_ (IV = Sn, Ge, Pb; VI = Se, Te, S) compounds crystallize in more distorted structures that are far from tetragonal, as shown in Figure 1a. Cu_2_SnSe_3_ is a kind of CBDL compound with diverse structural phases, which has been found and synthesized successfully, including in cubic, tetragonal, orthogonal, and monoclinic phases involving three variants [22]. Hu et al. [62] improved the TE transport behaviors of Cu_2_SnSe_3_ by enhancing the crystal symmetry of it via Mg-doping and intensifying the phonon scattering through the introduction of dislocations and nanoprecipitates. Similarly, Ming et al. [65] obtained a peak *zT* of 1.51 at 858 K in the Cu_2_Sn_0.82_In_0.18_Se_2.7_S_0.3_ compound through regulating the band structure and introducing multi-scale defects. In addition, a record-high *zT* of 1.61 was obtained at 848 K by Qin et al. [66] by constructing the intrinsic point defects, including high-dense stacking faults and endo-grown nanoneedles, to obstruct mid- as well as low-frequency phonons in Cu_2_SnSe_3_ compounds. Except for Cu_2_SnSe_3_, Cu_5_A_2_B_7_ (A = Si, Ge, Sn; B = S, Se, Te), with a centrosymmetric space group *C2/m*, is also a kind of distorted CBDL compound which has been considered to possess a non-centrosymmetric cubic structure, with the phase crystallized as *C-*centered, as shown in Figure 2a [91,92,93]. An undesirable characteristic of Cu_5_A_2_B_7_ compounds is that they represent metal-like behaviors, such as the carrier concentration and *κ* of Cu_5_Sn_2_Te_7_ at 300 K are 1.39 × 10^21^ cm^−3^ and 15.1 W·m^−1^·K^−1^, respectively [92]. Simultaneously, zinc atoms have been proven to be effective dopants for strengthening the semiconductor properties of Cu_5_Sn_2_Te_7_ compounds; Sturm et al. [93] introduced a zinc dopant into Cu_5_Sn_2_Se_7_ and Cu_5_Sn_2_Te_7_ compounds, which also supports this conclusion. Especially noteworthy is that the effect of zinc doping is not optimal, and the TE performance of the compound still needs further improvement.

Quaternary Cu_2_–II–IV–VI_4_ (II = Co, Mn, Hg, Mg, Zn, Cd, Fe; IV = Sn, Ge; VI = Se, S, Te) compounds with more complex tetragonal structures have also been widely studied. The distinguishing features of quaternary CBDL compounds are they possess a wider bandgap and a relatively lower carrier mobility compared with the ternary CBDL compounds [68,70,76,94,95,96,97,98,99]. Taking the orthorhombic enargite-type Cu_2_MnGeS_4_ as an example [95], the bandgap of it is ~1.0 eV in the initial phase while it only converts to 0.9 eV in the Cu_2.5_Mn_0.5_GeS_4_ by adjusting the ratio of Mn and Cu atoms. The large-cell Cu_10_B_2_C_4_D_13_ [100,101,102,103] (B = Ag, Cu; C = Co, Ni, Zn, Cu, Mn, Fe, Hg, Cd; Q = Sb, Bi, As; Q = Se, S) tetrahedrites have even more complex crystal structures, as shown in Figure 2b,c, respectively. The featured “PGEC” framework is also displayed in the Cu_12_Sb_4_S_13_ tetrahedrite, where the electric transmission is controlled by a CuS_4_ network and the thermal transmission is governed by a cavity polyhedral consisting of CuS_3_ and SbS_3_ groups [100]. In 2013, Lu et al. [102] achieved an enhanced *zT* of 0.95 at 720 K in Cu_12_Sb_4_S_13_ utilizing Zn-doping. Moreover, Li et al. [103] attained a high *zT* of 1.15 at 723 K in a porous Cu_12_Sb_4_S_13_–based material; a segmented single-leg device based on the material was successfully fabricated which realized a high conversion efficiency of 6% when the Δ*T* reached up to 419 K. Cu_26_P_2_Q_6_S_32_ [104,105,106,107,108] (P = V, Ta, Nb, W, Mo; Q = Ge, Sn, As, Sb) colusites are other large-cell examples, which possess 66 atoms in a crystal cell while the tetrahedrites possess 58 atoms. Therefore, the common characteristic of both is their inherent low *κ* derived from high structural inhomogeneity [108,109]. For instance, Guilmeau’s group [105] obtained the lowest *κ* of 0.4 W·m^−1^·K^−1^ at 300 K in the Cu_26_V_2_Sn_6_S_32_ colusite, which was attributed to the structural complexity of colusite and mass fluctuations among the Cu, V and Sn atoms. In 2018, they further elucidated the potential mechanism related to the fountainhead of intrinsically low *κ* for a colusite along with the influence of antisite defects and S-vacancies on carrier concentration [105,106].

**Table 1:** Thermoelectric transport properties of selected CBDL compounds.

Composition	*zT_max_*	*zT_ave_*	*α^2^σ* (μW·cm^−1^·K^−2^)	*κ_L_* (W·m^−1^·K^−1^)	*n*@RT (10^19^cm^−3^)	Synthesis Method *	Ref.
Cu_0.75_Ag_0.2_InTe_2_	1.24@850 K	0.47	7.26	0.3	1.11	M + HP	[23]
Polycrystalline CuGaTe_2_	1.40@950 K	0.43	8.9	0.45	0.11	M	[29]
Cu_0.7_Ag_0.3_Ga_0.4_In_0.6_Te_2_	1.64@873 K	0.73	5.22	0.24	0.007	M + HP	[26]
(CuInTe_2_)_0.99_(2ZnTe)_0.01_–0.1 wt% TiO_2_	1.47@823 K	0.50	12.93	0.45	6.01	M	[36]
Cu_0.89_Ag_0.2_In_0.91_Te_2_	1.60@850 K	0.49	8.81	0.36	0.07	M + SPS	[38]
Cu_7.9_In_8.1_Ga_0.3_Te_16_	1.22@850 K	0.51	11.92	0.55	7.03	M + SPS	[39]
Cu_0.7_Ag_0.3_GaTe_2_	1.00@750 K	0.57	12.26	0.68	4.8	M + HP	[40]
Cu_0.8_Ag_0.2_In_0.2_Ga_0.8_Te_2_	1.50@ 850 K	0.78	14	0.49	0.043	M + SPS	[41]
Cu_3_SbSe_4_+15 vol% β–Zn_4_Sb_3_	1.23@648 K	0.43	12.7	0.14	5.5	ST	[45]
Cu_3_Sb_0.96_Sn_0.04_Se_4_–5 wt% AgSb_0.98_Ge_0.02_Se_2_	1.23@675 K	0.50	13.8	0.54	8.72	M + SPS	[47]
Cu_2.8_Ag_0.2_Sb_0.95_Sn_0.05_Se_4_	1.18@623 K	0.36	9.54	0.27	12.0	MAH + SPS	[48]
Cu_2.85_Ag_0.15_SbSe_4_	0.90@623 K	0.52	10.98	0.66	0.57	M + SPS	[49]
Cu_3_SbSe_4_–4 wt% CuAlSe_2_	1.40@723 K	0.72	16	0.35	10	M + HP	[27]
Cu_0.92_Zn_0.08_FeS_2_	0.26@623 K	0.14	5.4	2.24	39.6	M	[52]
Cu_0.92_In_0.08_FeS_2_	0.35@723 K	0.19	4.7	0.79	41.2	M + SPS	[53]
Cu_0.88_Ag_0.12_FeS_2_	0.45@723 K	0.22	7.6	1.15	3.6	M + PAS	[54]
CuFe_0.94_Ge_0.06_S_2_	0.40@723 K	0.17	6	1.04	4.7	M + HP	[55]
Cu_2_Sn_0.9_In_0.1_S_3_	0.60@773 K	0.32	6.23	1.01	126	MA + SPS	[56]
Cu_2_Sn_0.85_Mn_0.15_S_3_	0.68@723 K	0.28	9.2	0.4	462	M + SPS	[57]
Cu_2_Sn_0.74_Sb_0.06_Co_0.2_S_3_	0.88@773 K	0.43	10.4	0.41	237	M + SPS	[58]
Cu_1.85_Ag_0.15_(Sn_0.88_Ga_0.1_Na_0.02_)Se_3_	1.60@823 K	0.50	12.75	0.28	91.8	M	[62]
Cu_1.85_Ag_0.15_Sn _0.9_In_0.1_Se_3_	1.42@823 K	0.38	9.70	-	73.4	SHS	[63]
Cu_1.85_Ag_0.15_Sn_0.9_1In_0.09_Se_3_/4% Ag_2_S	1.58@800 K	0.59	12.6	0.12	133.4	SHS + PAS	[64]
Cu_2_Sn_0.82_In_0.18_Se_2.7_S_0.3_	1.51@858 K	0.33	9.3	0.35	151	M	[65]
Cu_2_Sn_0.88_Fe_0.06_In_0.06_Se_3_–5 wt% Ag_2_Se	1.61@848 K	0.40	7.6	0.2	163	M + MA + HP	[66]
Cu_1.9_Ag_0.1_Ge_0.997_Ga_0.003_Se_3_	1.03@768 K	0.58	7.3	0.46	3.5	M + SPS	[60]
Cu_1.8_Ag_0.2_Ge_0.95_In_0.05_Se_3_	0.97@723 K	0.44	6.4	0.38	4.6	M + HP	[61]
Cu_11.7_Gd_0.3_Sb_4_S_13_	0.94@749 K	0.46	16	-	60.3	M + HP	[68]
Cu_11.25_Cd_0.75_Sb_4_S_13_	0.90@623 K	0.72	12.1	0.33	42	M + HP	[98]
Cu_11.5_Ni_0.5_Sb_4_S_13_+0.7 vol% AP	1.15@723 K	0.66	12.8	0.17	-	MA + SPS +	[103]
Cu_3_SbS_4_–9 wt% CuAlS_2_–1.5 wt% AgAlS_2_	1.30@773 K	0.77	16.1	0.72	42.2	M + HP	[28]
Cu_3_Sb_0.95_Sn_0.05_S_4_	0.72@623 K	0.37	11.3	0.85	41.4	MA + SPS	[50]
Cu_3_Sb_0.89_Bi_0.06_Sn_0.05_S_4_	0.76@623 K	0.38	13.98	0.78	74	MA + SPS	[51]
Cu_2.10_Cd_0.90_SnSe_4_	0.65@700 K	0.27	5.1	0.23	-	M + SPS	[70]
Cu_2_CoSnSe_4_	0.70@850 K	0.31	6.83	0.45	19	M + SPS	[76]
Cu_2_MgSn_0.925_In_0.075_Se_4_	0.42@700 K	0.17	5.8	-	14	M + SPS	[79]
Cu_2.1_(Fe_0.5_Mn_0.5_)_0.9_SnSe_4_	0.60@800 K	0.22	6.1	-	30	M + SPS	[77]
Cu_2.1_Fe_0.9_SnSe_4_	0.52@800 K	0.23	5.9	0.60	23	M + SPS	[78]
Cu_2_CdSnSe_4_	0.50@760 K	0.17	3.1	0.42	1.15	CS + M + SPS	[71]
Cu_2.1_Cd_0.8_SnSe_3.4_	0.65@723 K	0.27	6.96	0.42	-	ST + HP	[72]
Cu_1.7_Ag_0.3_CdSnSe_4_	0.80@688 K	0.43	6.5	0.37	-	MAH + SPS	[73]
Cu_2_CdSnSe_4_–CdSe	0.65@725 K	0.34	5.1	0.56	60	M + HP	[74]
Cu_26_Nb_2_Ge_6.0_S_32_	1.00@670 K	0.50	8	0.51	-	M + HP	[104]
Cu_26_V_2_Sn_6_S_32_	0.93@675 K	0.55	7.73	0.4	380	MA + SPS	[106]
Cu_26_Cr_2_Ge_6_S_32_	1.00@700 K	0.48	19.4	0.48	-	MA + SPS	[108]

* Herein, melting abbreviated to M, hot-pressing abbreviated to HP, spark plasma sintering abbreviated to SPS, microwave-assisted hydrothermal abbreviated to MAH, mechanical alloying abbreviated to MA, plasma-activated sintering abbreviated to PAS, solvothermal abbreviated to ST, self-propagating high-temperature synthesis abbreviated to SHS and colloidal synthesis abbreviated to CS.

## 3. Material Synthesis Recipes

**Figure 3:**
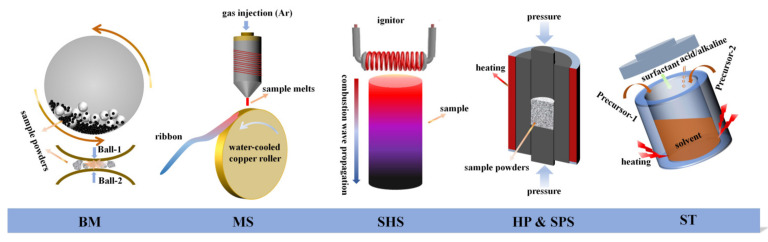
Schematically illustration of BM, MS, SHS, HP&SPS and ST.

The synthesis process accompanied by the research and development of the material is a crucial link in obtaining superior TE materials. Therefore, while the performance of TE materials have been improved by leaps and bounds, diverse techniques for synthesizing various TE compounds are also developing vigorously. As shown in Table 1, traditional technologies such as melting, the so-called solid-state reaction, are still widely used in the preparation of high-performance TE materials. Letting nature take its course, the successful application of non-equilibrium formulations, including high-energy ball milling (BM), melt spinning (MS), self-propagating high-temperature synthesis (SHS) and solvothermal (ST) technologies in the TE field provide more options for developing the new generation of TE materials with fine multi-scale microstructures. Simple schematic diagrams of several common synthesis and preparation technologies are shown in Figure 3.

High-energy ball milling, also known as mechanical alloying, has been widely adopted to assist in, or directly, synthesize TE compounds with multi-dimensional structures [111,112,113,114,115,116,117,118]. For instance, Nautiyal et al. [117] synthesized a series of polycrystalline Cu_2_SnS_3_, Cu_2_ZnSe_4_ and Cu_2_ZnSnS_4_ TE compounds through MA, which proved that the introduction of nanostructures into the material stabilized the disordered phase structure at low temperatures was conducive to optimizing the TE transport performance of the material. The mechanism of high-energy reaction is achieved by using the inertia between the grinding balls to cause a high-energy impact on the material particles, resulting in cold welding, fracture and re-welding between the particles, leading to further crushing [114]. In addition, most of the BM process involves dry grinding in protective gas to ensure that the collision energy among balls can be effectively applied to the ground powders, and sometimes ethanol and other solvents are used as grinding media. After BM, the fine structure and even nano-powders existing in the material can effectively enhance the phonon scattering and significantly reduce the *κ*. BM has the advantages of high synthesis efficiency, easy operation, high cost-efficiency and the ability to synthesize thermoelectric materials in large quantities. It is usually used to produce multi-dimensional structure [115], synthetic compound [111,116,117] and mix composites [118] in the TE industry.

Melt-spinning technology is an effective approach to achieve rapid solidification by injecting a molten alloy flow into a rotating and internally cooled roller [119], as shown in Figure 3. When the melt contacts the roller, the melt will undergo rapid solidification or even amorphous transformation accompanying the rapid transfer of heat and will be produced in the form of thin strips or ribbons [119,120,121]. The microstructure, that depends on local temperature and cooling rate, can be easily controlled by adjusting the machining parameters in the process of MS [119,122]. Previous studies have shown that a large number of refined microstructures and nano-grains can be introduced in TE compounds by MS, such as SnTe, BiSbTe, PbTe and skutterudite, etc., [120,123,124,125]. In 2019, Zhao’s group [121] successfully prepared Cu-Te alloy ribbons with nanocrystalline structures using MS, and achieved the lowest *κ* of 0.22 W·m^−1^·K^−1^ in the Cu_2_SnSe_3_–based composite. 

The self-propagating high-temperature synthesis starts with the heating of a small part of the sample at a point, and then the combustion wave spreads along the material to gradually realize the synthesis of the material in an extremely short amount of time [64,126], as shown in Figure 3. In 2014, Su et al. [126] successfully applied SHS to the preparation of various TE compounds for the first time, including Cu_2_SnSe_3_, CoSb_3_, Bi_2_(Te, Se)_3_, SnTe, Mg_2_(Sn, Si), etc. As the combustion wave spreads across the whole sample, it plays a role in purifying the material and maintaining its stoichiometry [126,127]. The most attractive aspect of SHS is its rapid one-step process, which can be expanded and completed with minimal energy. This feature makes it popular in the synthesis of a variety of CBDL compounds [63,64,127,128]. The main shortcoming of the self-propagating high-temperature synthesis process is that the reaction is so rapid that the sintering size of the sample is difficult to control, requiring secondary processing to ensure the quality of the materials [64,126]. For subsequent measurements and characterizations, dense block TE materials are generally manufactured using sintering technology, including HP and SPS (also known as plasma-activated sintering (PAS)). In most cases, the procedure of sintering is the last step of fabrication, as shown in Table 1, which can strengthen the densification of products and further purify the phases. 

In addition, hydrothermal as well as solvothermal reactions are very efficient approaches to preparing refined materials with controllable dimensions and morphologies through the chemical synthesis process [42,45,48,129,130,131,132,133]. In the process of ST, the stoichiometric precursor material required for the synthetic material is first dissolved in the aqueous solution, and then the internal reaction conditions, such as the pressure, pH value and additive concentration, are strictly controlled to make it react in a sealed autoclave [130,131,134,135,136]. Although the operation is more complex compared to the physical methods mentioned above, controllable thermoelectric compound nanostructures can be synthesized through a wet process, which has the advantages of a low synthesis temperature and fine grain size. It is also worth noting that some the morphologies and sizes of the products can be greatly modified by external conditions, such as the ultrasonic mixture pretreatment time, and the reaction temperature and time [131]. For instance, Wang et al. [136] synthesized the monodispersed Cu_2_SnTe_3_ nanocrystals (~25 nm) using hot-injection synthesis for the first time, in which the Te precursor was selected by dissolving TeO_2_ in 1–dodecanethiol and the reaction solvent was a Cu–Sn complex solution. Moreover, Wei et al. [135] synthesized Cu_3_SbSe_4_ hollow microspheres dispersed with TiO_2_ by a procedure of microwave-assisted hydrothermal synthesis. One advantage of chemical synthesis is that it can control the doping of foreign ions and optimize the grain orientations of nanostructures, which has an important impact on adjusting the carrier concentration and improving phonon scattering [43,129,132,135,136].

**Figure 4:**
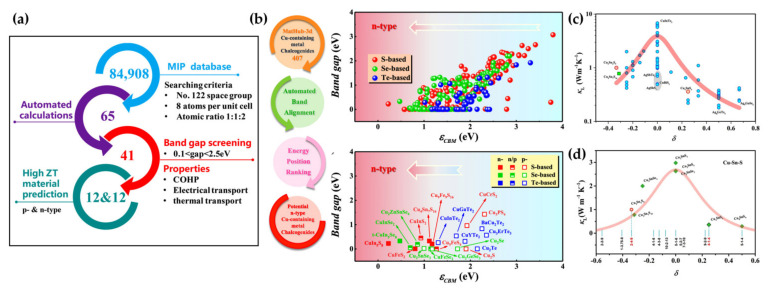
(**a**) The high-performance thermoelectric material screening workflow for ternary compounds ABX_2_ with diamond-like structures, reprinted with permission from ref. [139], copyright 2019 American Chemical Society; (**b**) workflow of the HTP screening process in Cu–containing metal chalcogenides, reprinted with permission from ref. [140], copyright 2022 American Chemical Society. Room temperature *κ_L_* varying with number mismatch in (**c**) ternary Cu– and Ag–based chalcogenides; and (**d**) Cu–Sn–S compounds, reprinted with permission from ref. [145], copyright 2020 the Springer Nature.

In recent years, additive manufacturing [137,138] and machine learning [14,139,140,141,142,143,144], as emerging intelligent industries, have gradually entered the thermoelectric field, which opens a novel and convenient means to exploring multi-phase space. Referring to diverse indicators closely related to material properties, a series of high-performance CBDL compounds have been discovered. For instance, Zhang’s group [139] investigated and predicted the electronic structures and the TE transport behaviors of ABX_2_ materials using a high-throughput (HTP) framework, as shown in Figure 4a. Taking the energy position of the band edge as an indicator, Chen’s team [140] verified the HTP strategy with the bandgap as an indicator by screening out the potential high-performance *n-*type TE compounds from Cu-containing chalcogenides, as shown in Figure 4b. In addition, Shi et al. [145] proposed a new performance indicator, shown in Figure 4c,d, for guiding the discovery of TE compounds with low *κ*. The new indicator referred to the number mismatch (δ) between anions and cations. It should also be noted that since the difference of atomic mass was not considered, the indicator was applicable to compound families with the same elements but different compositions. It was well demonstrated in the Cu-Sn-S systems shown in Figure 4d.

## 4. Strategies for Optimizing the TE Performances of CBDL Compounds

There are two main basic principles for achieving high-performance TE materials, one of which is to maximize the *PF* while the other is to minimize the *κ_L_*. One of the typical characteristics of CBDL compounds is that the highly degenerated valence band results in the compound possessing a high Seebeck coefficient. The common defect for most CBDL compounds is that they generally have low carrier concentrations at low temperatures and high *κ_L_* in their initial form. Therefore, trying to promote or maintain *PF* is the critical issue in the development of high-performance CBDL compounds while reducing the *κ_L_*.

### 4.1. Several Representative Strategies for Enhancing Power Factor

#### 4.1.1. Optimization in Carrier Concentration

As most TE materials have an optimal carrier concentration in the range of 10^19^ to 10^21^ cm^−3^, one of the most common approaches to maximizing the PFs of TE materials is tuning the carrier concentration [4,5]. For optimizing the carrier concentration in CBDL compounds, a quantity of impurities with different functions has been introduced into pristine compounds. Successful cases among Cu(In, Ga)_1__−x_N_x_Te_2_ (M = Ag, Zn, Ni, Mn, Cd, Hg, Gd) [23,34,146,147,148,149,150,151], Cu_1__−x_Fe_1+x_S_2_ [152], Cu_3_Sb_1__−x_N_x_Se_4_ (N = As, Zr, Hf, Al, In, Sn, Ge, Bi, La) [42,43,113,115,132,153,154] and Cu_2_Cd_1__−x_In_x_SnSe_4_ [155] have demonstrated that doping towards a higher charge-carrier density can effectively improve the electrical performances of the compounds. In addition, introducing vacancies is also another available approach to optimizing the electrical transport properties as well as minimizing the *κ_L_*. On the one hand, as the most common form of *p-*type doping, Cu vacancy has been widely created in CBDL compounds owing to the small formation energy of defects, as seen in Cu_12__−x_N_x_Sb_4_S_13_ (N = Cd, Mn, Ge, Fe, Co, Sn, Ni, Bi, Zn) [98,156], Cu_1__−x_(In, Ga)Te_2_ [40,157,158] and Cu_3__−x_SbSe_4_ [159]. On the other hand, it is feasible to use anion vacancies for donor doping, as displayed in Cu_2_ZnGeSe_4__−x_S_x_ [160], CuFeS_2__−x_ [161], Cu_12_Sb_4_S_13__−x_Se_x_ [100] and Cu_2_FeSnS_4__−x_ [162].

#### 4.1.2. Modulation Doping

**Figure 5:**
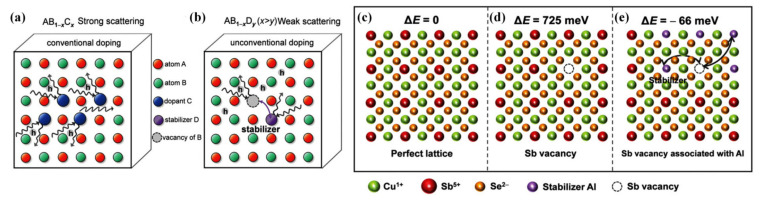
Schematic diagrams of (**a**) conventional; and (**b**)unconventional doping; Cu_3_SbSe_4_ with (**c**) perfect lattice; (**d**) Sb vacancy; and (**e**) Sb vacancy surrounded by Al as a stabilizer. Reprinted with permission from ref. [27], copyright 2022 Wiley-VCH GmbH.

It should be pointed out that the effect of traditional doping by substituting host atoms by alien ones is fettered by the solubility limit, and worse still, it is easy to cause intense charge-carrier scattering at room temperature, resulting in a loss of electrical transport performance [7]. Compared with traditional doping, modulation doping can effectively avoid the above problems. It is usually designed as a composite composed of two kinds of nanoparticles, and only one of them contains a doping agent [86]. Recently, an unconventional doping (UDOP) strategy was proposed by Zhou et al. [27,28,163] supported this view, where the increase in the vacancies concentration was obtained from an Sb vacancy stabilized by Al rather than alien atoms. Combined with an optimized hole concentration (3.1 × 10^20^·cm^−3^) and a maintained carrier mobility, a considerably high average *PF* of 19 µW·cm^−1^·K^−2^ was obtained in the temperature range of 300–723 K [27]. In contrast to the conventional doping method (Figure 5a), the carrier concentration and carrier mobility decouple by vacancies in the route of UDOP (Figure 5b). In other words, it can be considered that in the purposeful doping process, the doping additive itself does not provide carriers, but acts as a “stabilizer” of the cationic vacancy (Figure 5c–e), which actually offers additional holes for *p-*type conductive semiconductors. It has been proved that the modulation-doping strategy can be used to not only improve the *PF* of CBDL compounds, but also to maintain the carrier mobility of various compounds requiring a high carrier concentration.

#### 4.1.3. Pseudocubic Structure

**Figure 6:**
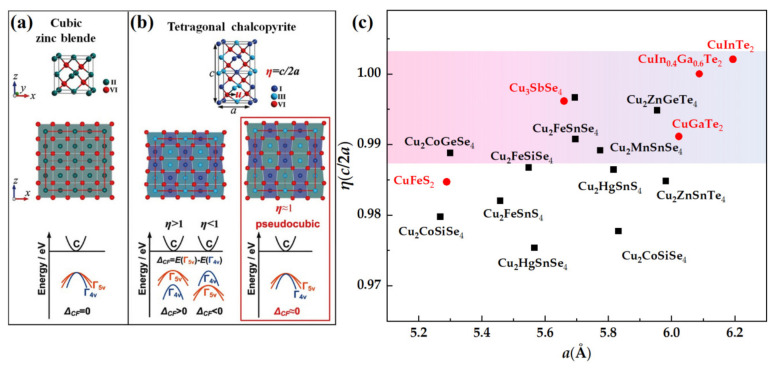
Band convergence in (**a**) cubic zincblende structure; and (**b**) pseudocubic ternary chalcopyrites, reprinted with permission from ref. [31], copyright 2014 WILEY-VCH Verlag GmbH & Co. KGaA, Weinheim. The *c* and *a* are the lattice constants. Γ*_4v_* is a nondegenerate band, and Γ*_5v_* is a doubly degenerate band. ∆*_CF_* is the crystal field-induced energy split at the top of the Γ*_4v_* and Γ*_5v_* bands; (**c**) distortion parameter *η* as a function of the lattice parameter *a*, reprinted with permission from ref. [32], copyright 2018 Science China Press and Springer-Verlag GmbH Germany, part of Springer Nature.

Apart from obtaining an optimal carrier concentration, the regulation of *PF* is also linked with the electronic band structure [5,7,8]. The high band convergence (*N_v_*) originating from high symmetric crystal structures is beneficial for obtaining large α and high σ. Similarly, the CBDL compounds derived from the high-symmetry cubic phase, ensures that they possess highly degenerate valence bands [18,32,33]. In particular, Zhang et al. [31] proposed a pseudocubic strategy were the *PF* could be optimized to the greatest extent by pruning the band split-off, which was also considered as an efficient approach to exploring and screening high-performance non-cubic TE compounds. As shown in Figure 6a,b, when the valence band-splitting energy Δ*_CF_* approximates to zero, this means that the distortion parameter *η* = *c/*2*a* approaches one; in other words, the bands are in a degenerate state at this time, which can trigger the maximum *PF*. The pseudocubic approach, also known as the unity-*η* rule (*η* = *c/*2*a*), has been successfully applied to screen out high-performance tetragonal CBDL compounds [23,35,76,97]. For instance, Li et al. [97] found that the Δ*_CF_* of Cu_2_ZnSnSe_4_ could be appropriately tuned by applying the proper strain, which provides an alternative way to improve the thermoelectric properties of the compound. As a systematic strategy, the unity-*η* rule is used to qualitatively guide the evaluation and manipulation of TE diamond-like lattices. For instance, the distortion parameter *η*, as a function of the cell parameter *a*, for tetragonal diamond-like chalcogenides [32] is shown in Figure 6c. It should be noted that the pseudocubic approach is limited to low-symmetry material with an ideal bandgap and a low *κ_L_* [7]. 

#### 4.1.4. Softening *p-d* Hybridization

**Figure 7:**
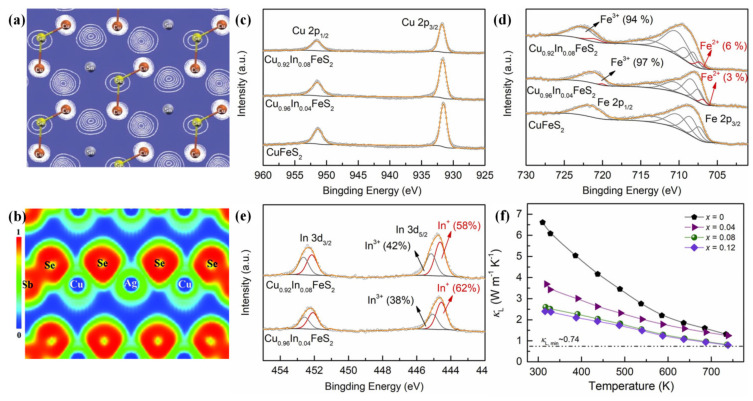
(**a**) Schematic diagrams of the partial charge density of the states close to upper valence-band in Cu_2_SnSe_3_ on (100) crystal face, reprinted with permission from ref. [25], copyright 2010 American Chemical Society; (**b**) the calculated electron-localization function of Ag-doped Cu_3_SbSe_4_ on (101) crystal face, reproduced from ref. [49], copyright 2019 the Royal Society of Chemistry. The X-ray photoelectron spectra (XPS) of (**c**) Cu 2*p*; (**d**) Fe 2*p*; and (**e**) In 3*d* for the Cu_1__−x_In_x_FeS_2_ samples; and (**f**) the *κ_L_* of Cu_1__−x_In_x_FeS_2_ samples, reproduced from ref. [53], copyright 2022 Elsevier Ltd.

It is well known that the *p-d* hybridization in CBDL compounds is very strongly attributable to the quite small separation energy among the atomic levels of chalcogen *p-*orbitals and Cu-3*d* states [91,99,164]. For most CBDL compounds, the electric transport channel (mostly the valence band maxima, VBM) is regarded as being constructed by Cu-X bonds [18,22]. The chemical bonding and the electronic structure in TE materials are closely linked to their internal charge carriers and phonon transport behaviors. Therefore, the regulation of *p-d* hybrid strength potentially serves as an adjustable critical parameter in adjusting the properties of CBDL compounds. Taking Cu_2_SnSe_3_ [25] as an example in Figure 7a, the VBM is mostly occupied by the *p-d* hybridization from Cu-Se bonds, which acts as the charge-conduction pathway as well as a structural retainer. In contrast, the *p-*orbitals of Sn atoms contribute little to the occupied states while the conductive band is primarily dominant. Similarly, the Cu-X conduction channel has also been demonstrated in some other CBDL compounds, such as CuGaS_2_ [164], Cu_3_SbSe_4_ [24], Cu_2_CdZnSe_4_ [70], etc. The Cu_3_SbSe_4_ diamond-like compound with a small bandgap is also an example influenced by the strong relativistic orbital-contraction effect [164,165]. Softening *p-d* hybridization, as an active strategy, has been adopted to synergistically improve the electronic and thermal transport performance of Cu_3_SbSe_4_ via Ag-doping [49,166], as shown in Figure 7b. Zhang et al. [49] discovered that the *PF* of Cu_3_SbSe_4_ was significantly enhanced by changes in the bandgap and the density of states caused by the softening of *p-d* hybridization, which, accompanied by Ag-doping, induced large strain fluctuations in some local structural distortions and resulted in greatly reduced *κ_L_*. In addition, Ge et al. [53] introduced an abnormally high concentration of indium in CuFeS_2_ compound, as shown in Figure 7c–e; the indium was not fully ionized to In^3+^ cation when on the Cu sublattice and existed mainly in the In^+^ oxidation state. The latter, with 5*s*^2^ lone-pair electrons, could cause strong local bond distortions, thereby softening the In-S and Cu-S bonds and introducing localized low-frequency vibrations [89]. Therefore, a low *κ_L_* value of 0.79 W·m^−1^·K^−1^ (Figure 7f) and a high *zT* value of 0.36 were recorded at 723 K in Cu_1__−x_In_x_FeS_2_ samples.

### 4.2. Strategies for Reducing Lattice Thermal Conductivity

#### 4.2.1. Point-Defect Scattering

**Figure 8:**
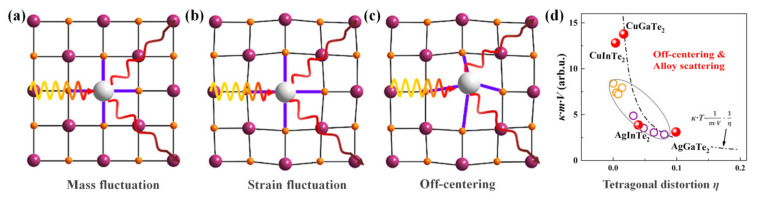
Schematic diagrams of (**a**) mass fluctuation; (**b**) strain fluctuation; (**c**)off-centering effect in phonon transport; and (**d**) relationship between tetragonal distortion and thermal conductivity for different Ag-based and Cu-based diamondoid compounds, the *V* is crystal volume, *m* is the formula weight. Reprinted with permission from ref. [151], copyright 2023 American Chemical Society.

In the thermoelectric field, defect and nanostructuring engineering have been widely adopted to optimize the thermoelectric performance enhancement of TE materials, especially the dislocations and nanostructured interfaces which involve the scattering of low- and mid-frequency phonons, have received more attention [6]. In the process of improving the TE performance for CBDL compounds, the existence of point defects plays a more important and beneficial role in phonon scattering than in affecting the electrical behavior. There are two main types of influence on *κ_L_* originating from point defects in TE materials: the mass fluctuation (Figure 8a) and strain field fluctuation (Figure 8b) among the host and guest atoms. Shen et al. [40] testified that substitutional defects of *Ag_Cu_* in CuGaTe_2_ could reduce the *κ_L_* more efficiently than substitutional defects of *Zn_Ga_* or *In_Ga_* at the equivalent concentration, which was attributable to the larger mass fluctuation. When the dominant point defects are vacancies, the types of scattering inflected by the strain and mass fluctuations can be maximized [5]. Thus, the compounds with an intrinsic high concentration of cation vacancies, such as In_2_Te_3_ and Ga_2_Te_3_, were introduced in CuGaTe_2_ to depress the *κ_L_* of the matrix phase by constituting solid solutions [80]. Additionally, an elaborate investigation about the room temperature κ for cation-substituted Cu_2_ZnGeSe_4__−x_S_x_ compounds displayed a reduction of 42% for *κ_L_*, where the reduction caused by mass contrast accounted for 34% and the remaining 8% was caused by strain fluctuations [160]. In their latest study, Xie et al. [151] observed the off-centering effect (Figure 8c) of an Ag atom by investigating the thermal transmission behaviors in Cu_1_–*_x_*Ag*_x_*GaTe_2_ as well as in CuGa_1_–*_x_*In*_x_*Te_2_. It is obvious that the off-centering behavior of the Ag atom means a new phonon scattering mechanism is brought about by point defects, where the Ag-alloyed solid solutions resulted in an extremely low *κ_L_*, which was attributed to crystallographic distortion and extra-strong acoustic-optical phonon scattering, as shown in Figure 8d. Moreover, it can also be seen that a modified Klemens model was developed by integrating the off-centering effect and alloy-scattering with the crystallographic distortion parameter (*η*), which can be used as an indicator to predict the *κ* of diamondoid solid solutions.

#### 4.2.2. Nanostructure Engineering

**Figure 9:**
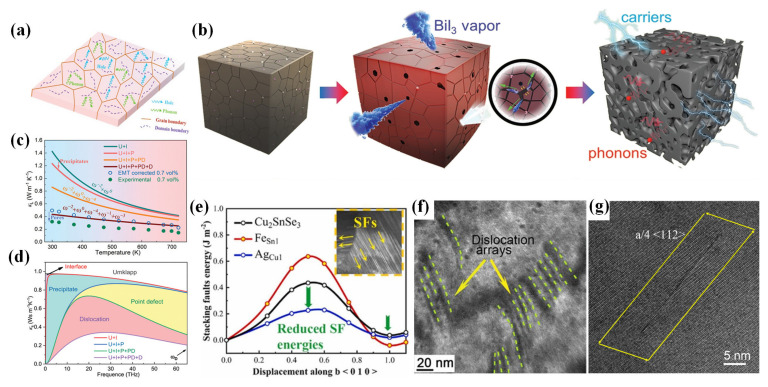
(**a**) Schematic illustration of the transport behaviors for phonons and holes in CuGaTe_2_, reprinted with permission from ref. [26], copyright 2019 WILEY-VCH Verlag GmbH & Co. KGaA, Weinheim; (**b**) schematic illustration showing the formation of a porous network during BiI_3_ sublimation; (**c**) *κ_L_* of sample with 0.7 vol% AP, which took Umklapp process (U), porous interfaces (I), precipitates (P), point defects (PD), dislocation cores (DC), and strains (DS, D = DC + DS) into account; (**d**) frequency-dependent accumulative reduction in the lattice thermal conductivity of the EMT-corrected sample with 0.7 vol% AP due to various scattering mechanisms. Reprinted with permission from ref. [103], copyright 2021 Wiley-VCH GmbH; (**e**) calculated generalized stacking fault energies as a function of normalized Burger’s vector *b* <010> in Cu_2_SnSe_3_–based system, the insert was the high-dense stacking faults (SFs) in (Fe, Ag, In)-doped Cu_2_SnSe_3_. Reprinted with permission from ref. [66], copyright 2022 Elsevier Ltd. High-dense SFs in (**f**) (Ag, Ga, Na)-doped (reprinted with permission from ref. [62], copyright 2021 Wiley-VCH GmbH); and (**g**) Ni-doped (reprinted with permission from ref. [168], copyright 2021 American Chemical Society) Cu_2_SnSe_3_.

Controlling the nanostructures of TE materials is also an effective approach to enhancing phonon scattering through realizing an all-scale hierarchical architecture in TE materials. Zhang et al. [26] adopted a quinary alloy compound system with a complex nanosized strain-domain structure in CuGaTe_2_ (Figure 9a), which made the room-temperature *κ* decline from 6.1 W·m^−1^·K^−1^ for the initial compound to 1.5 W·m^−1^·K^−1^ for the Ag and In co-doped sample. Wang et al. [167] achieved low *κ* values of 0.491 W·m^−1^·K^−1^ and 0.481 W·m^−1^·K^−1^ in Cu_3_Sb_0.92_Sc_0.08_Se_4_ and Cu_3_Sb_0.92_Y_0.08_Se_4_ at 623 K, respectively, with a constructed multiscale heterostructure. In 2021, Hu et al. [103] designed pore networks for tetrahedrite Cu_12_Sb_4_S_13_–based TE materials using a BiI_3_ sublimation technique, as shown in Figure 9b, which led to a hierarchical structure which contained pores, pore interfaces, point defects, and granular precipitates. The effect of various scattering mechanisms on phonon-transport behaviors for Cu_12_Sb_4_S_13_–based samples are shown in Figure 9c,d. First, the existence of specially designed pores and pore interfaces reduced the *κ_L_* of samples with 0.7 vol% annealed pores (AP) by about 36%. Furthermore, Cu_1.8_S precipitates, point defects involved Ni-alloying and Bi-doping, dislocations, the solid solution of impurity Cu_3_SbS_4_ phase as well as volume expansion also contributed to the reduction of *κ_L_* because they realized full-scale phonon scattering in the TE sample. Consequently, a ~72% reduction in the *κ_L_* was obtained for samples with 0.7 vol% AP with the addition of a small amount of BiI_3_. Moreover, previous works demonstrated that high-density stacking faults (SFs) could be realized in doped Cu_2_SnSe_3_ [62,66,168], as shown in Figure 9e–g, which also caused strong scattering of phonons as a phonon-scattering center. In addition, solvothermal synthesis [43,134,135,153] and ball milling [113,115] are effective and convenient approaches to constructing nanostructures for TE materials. 

#### 4.2.3. Nanocomposite

**Figure 10:**
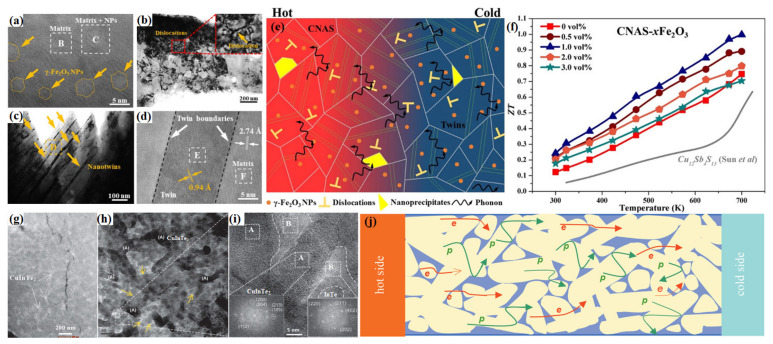
Microstructure of the Cu_11.5_Ni_0.5_Sb_4_S_13_–1.0% Fe_2_O_3_ sample including: (**a**)HRTEM image; (**b**) dislocation; (**c**) nanotwins; and (**d**) HRTEM images of the area D; (**f**) schematic diagram of phonon scattering in γ-Fe_2_O_3_ dispersed Cu_11.5_Ni_0.5_Sb_4_S_13_ (CNAS), (**e**) *zTs* for all CNAS-*x*Fe_2_O_3_ samples. Reprinted with permission from ref. [175], copyright 2020 American Chemical Society; (**g**) high-angle annular dark field; (**h**) high-resolution TEM image; (**i**) magnified TEM image and the fast Fourier transform of the CuInTe_2_:23 wt% InTe bulk sample; (**j**) schematic illustrating of the transport in both the phonons-p and electrons-e. Reprinted with permission from ref. [39], copyright 2020 WILEY-VCH Verlag GmbH & Co. KGaA, Weinheim.

Compositing with uniformly dispersed nanoinclusions, secondary phases or nanoparticles has been widely considered as a predominant and effective strategy to optimize TE performance in CBDL compounds [27,47,67,118,128,169,170,171,172]. Nanoparticles (NPs) introduced in composites can be effectively used as intermediate frequency phonons scatter centers and diminish *κ_L_* [5]. Sun et al. successfully incorporated ZnO [173] and Nb_2_O_5_ [174] NPs into the grain boundaries of Cu_11.5_Ni_0.5_Sb_4_S_13_ compounds via mechanical alloying and spark plasma sintering, respectively, and the both composites achieved a reduced *κ* and high *zTs*. In our previous work, we also introduced graphene nanosheets or SnTe NPs into Cu_3_SbSe_4_ through ball milling and realized the optimization of thermoelectric properties. Hu et al. [175] obtained a relatively low *κ* of 0.9 W·m^−1^·K^−1^ at all temperatures in Fe_2_O_3_–dispersed Cu_12_Sb_4_S_13_ tetrahedrite via the combination of nanostructuring and defect engineering (Figure 10a–e). As shown in Figure 10a–d, dislocations along with diverse nanostructures, such as NPs, nanotwins and nanoprecipitates, were introduced into Cu_11.5_Ni_0.5_Sb_4_S_13_ by compositing magnetic *γ*-Fe_2_O_3_ NPs, which realized all-scale hierarchical phonon scattering in the samples, making the *zT* reached up to ~1.0 (Figure 10f). For reducing *κ_L_*, Li et al. [39] synthesized CuInTe_2_–based compounds with in-situ formed InTe nanostrips, which wrapped the nanodomains (Figure 10g–j) and resulted in the reduction of *κ_L_* by a factor of ~2 compared to parent compound. It is notably anticipated that the content, dimensions and especially distribution of nano-additives in composites have an important impact the effective regulation of TE performances.

#### 4.2.4. Lattice Softening Effects

**Figure 11:**
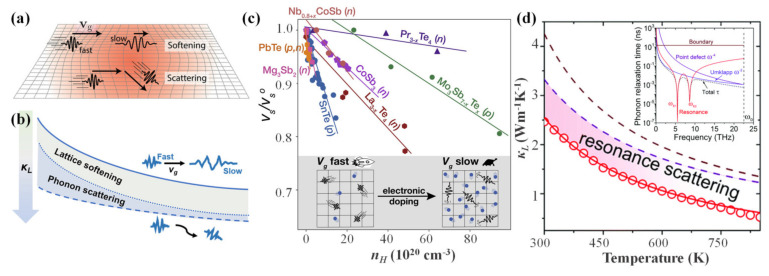
(**a**) Schematic illustration of lattice-softening effects and phonon scattering originated from internal-strain fields. Reprinted with permission from ref. [179], copyright 2019 WILEY-VCH Verlag GmbH & Co. KGaA, Weinheim; (**b**) schematic illustration of lattice-softening in Cu_2_Se. Reprinted with permission from ref. [177], copyright 2022, American Chemical Society; (**c**) sound velocities plotted against measured Hall charge-carrier concentration for SnTe, PbTe, Nb0_.8+x_CoSb, CoSb_3_, La_3__−x_Te_4_, Pr_3__−x_Te_4_, and Mo_3_Sb_7_. Reprinted with permission from ref. [180], copyright 2021 Elsevier Inc.; and (**d**) contribution of distinct scattering mechanism to the *κ_L_* of Cu_0.8_Ag_0.2_InTe_2_. Here the U, B, P and R represent Umklapp scattering, grain-boundaries scattering, point-defect scattering, and phonon-resonance scattering, respectively; the insert shows the calculated phonon relaxation times *τ* versus phonon frequency *ω* for Cu_0.8_Ag_0.2_InTe_2_ with different scattering mechanisms. Reprinted with permission from ref. [38], copyright 2020 The Royal Society of Chemistry.

The internal strain fluctuation induced by lattice defects, such as nanoprecipitates and dislocations, can locally shift the phonon frequencies in the TE material, which in principle can bring about lattice-softening accompanied by phonon scattering owing to changes in phonon speed, as shown in Figure 11a. In several cases, improvements in TE performance ascribed to lattice-softening through the introduction of vacancies or alloying have been presented [176,177,178], such as SnTe with AgSbTe_2_ alloying, and the lattice-softening effect in Cu_2_Se, as shown in Figure 11b. In 2019, Hanus et al. [179] authenticated that the changes of thermal transport behavior in the PbTe system were attributable to the lattice-softening through alloying or lattice defects, and pointed out that the modulation of lattice stiffness had a significant impact on the phonon transport in some states. In addition, Muchtar et al. [176] introduced lattice-softening into SnTe by inserting Ti and Zr atoms, which effectively suppressed the phonon group velocities and reduced the *κ*. Moreover, Snyder et al. [180] found the lattice-softening effect induced by charge-carrier-mediated in several high-performing (*zT* > 1) TE materials (such as SnTe, PbTe, Nb0_.8+x_CoSb, etc.) contributed more than 20% to *zT*. Simultaneously, the results shown in Figure 11c indicate that a strong dependence of sound velocities *v_s_* on Hall charge-carrier concentration *n_H_* was observed in each compound in which the measured *v_s_* significantly decreased with increasing *n_H_*. Lattice-softening effects also have been successfully used to improve the TE performances of CBDL compounds. Pöhls et al. [181] demonstrated that the Li-induced phonon-softening effect was feasible to enhance the TE performance of chalcopyrite CuGaTe_2_. Xie et al. [38] obtained an extremely low *κ_L_* of 0.47 W·m^−1^·K^−1^ at 850 K in Ag-doped CuInTe_2_ compound that was attributed to strong interactions among low-frequency optical phonons derived from the weakened Ag-Te bonds, as shown in Figure 11d.

### 4.3. Synergistic Regulation

#### 4.3.1. Entropy Engineering

**Figure 12:**
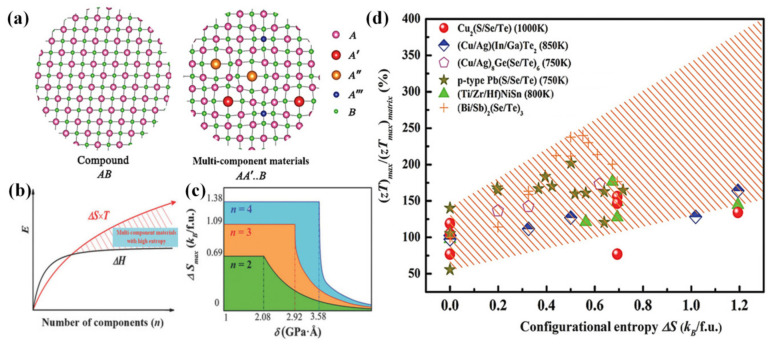
(**a**) Schematic diagram of the lattice framework in multicomponent materials compared to a simple binary compound; (**b**) schematic diagram of the entropy engineering with multicomponent TE materials; (**c**) the maximum configurational entropy (in units of *k_B_* per formula unit) as a function of a material’s solubility parameter δ for given multicomponent TE materials, where *n* is the number of components; and (**d**) plots of maximum *zT* versus the configurational entropy in several selected TE systems. Reprinted with permission from ref. [15], copyright 2017 WILEY-VCH Verlag GmbH & Co. KGaA, Weinheim.

In the process of optimizing the electrical and thermal transport properties of TE materials, it is never just to adjust one of them individually. To some extent, the above optimization process can realize the decoupling of electron and phonon transmission. Entropy engineering provides a new pathway to synergistically optimize the electrical, thermal, and mechanical properties for promoting the development of CBDL compounds [15,41,90,182,183]. Through synergistic regulation, Xie et al. [41] achieved a maximum *zT* of 1.5 at 850 K in the quinary (Cu_0.8_Ag_0.2_)(In_0.2_Ga_0.8_)Te_2_ compound, in which Ga-substituted In and Ag-substituted Cu effectively optimized the electrical and thermal transport properties, respectively. In addition, Cai et al. [183] obtained a high *zT* of 1.02 in CuInTe_2_ compound, which was attributed to the reduction of *κ* by devising a high-entropy structure as well as by improving the carrier mobility by one order of magnitude. In many cases long before that, Liu et al. [15] utilized the entropy attribute as the comprehensive gene-like performance indicator to screen and devise TE materials with high *zT*. As can be seen in Figure 12a,b, a special example can be noted that when multi-component alloy elements are adopted in compounds, the configurational entropy can especially be changed. For a given multi-component material, the maximum entropy lies on the solubility parameter *δ* of the whole material, which is linked to the mismatch of the atomic radius, shear modulus and lattice constant in the material, as shown in Figure 12c. Instructing with *δ*-criterion (Figure 12d), representative multi-component (Cu/Ag)(In/Ga)Te_2_–based CBDL compounds with *zTs* approaches to 1.6 were screened out owing to the optimization of entropy.

#### 4.3.2. Progressive Regulation Strategy

**Figure 13:**
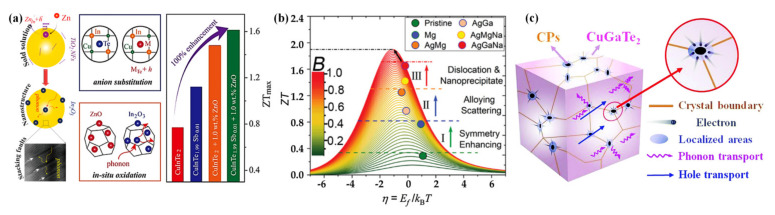
(**a**) Synergistic strategies of point defects and microstructure engineering in CuInTe_2_. Reprinted with permission from ref. [30,36], copyright 2015 Elsevier Ltd. All rights reserved and 2016 WILEY-VCH Verlag GmbH & Co. KGaA, Weinheim; (**b**) quality factor analysis on the relationship of chemical potential *η* versus *zT* in Cu_2_SnSe_3_–based compounds. Reprinted with permission from ref. [62], copyright 2021 Wiley-VCH GmbH; and (**c**) schematic diagram illustrating various phonon scattering mechanisms and the electron localized region near carbon particles (CPs) within the CuGaTe_2_+*x* wt% CPs sample. Reprinted with permission from ref. [135], copyright 2020 The Royal Society of Chemistry.

The progressive regulation strategy can be realized via integrating point defects and microstructure engineering. Luo et al. [30,36] successfully acquired high-performance CuInTe_2_ compounds by integrating the cation/anion substitution and in-situ oxidation, as shown in Figure 13a. Taking the in-situ substitution reaction between CuInTe_2_ and ZnO additive as a case [36], the priority generation of acceptor defects $Zn_{In}^{-}$ significantly optimized the *PF* while the In_2_O_3_ nanoinclusions incurred by the in-situ reaction led to a low *κ* of CuInTe_2_. Through triple doping in Cu_2_SnSe_3_, Hu et al. [62] obtained an excellent *zT* of 1.6 at 823 K in cubic Cu_1.85_Ag_0.15_(Sn_0.88_Ga_0.1_Na_0.02_)Se_3_ and a decent *zT_ave_* of 0.7 from 475 to 823 K in Cu_1.85_Ag_0.15_(Sn_0.93_Mg_0.06_Na_0.01_)Se_3_ via synergistic effects. As shown in Figure 13b, during the management process from the initial phase to (Ag, Ga, Na)-doped Cu_2_SnSe_3_, the gradually improved *zT* originated from symmetry enhancing, alloying scattering and dislocation/nanoprecipitate construction, respectively. Similarly, synergistically optimized CuGaTe_2_ [135] (Figure 13c), Cu_3_SbSe_4_ nanocrystals with Cu_2__−x_Se in-situ inclusions [48], CuIn_1__−x_Ga_x_Te_2_:yInTe with in situ formed nanoscale phase InTe [39], Cu_2_SnSe_3_ with CuInSe_2_ alloying [184], *etc*, demonstrated that the progressive and collaborative optimization strategies have been widely applied in CBDL materials.

## 5. CBDL-Based TE Devices

**Figure 14:**
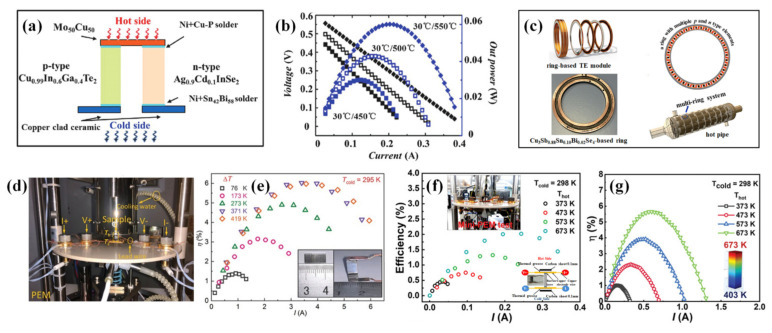
(**a**) Schematic diagram of the fabricated diamond-like module, and (**b**) plots of output voltage and power versus current for TE module based on diamond-like materials. Reprinted with permission from ref. [185], copyright 2018 The Royal Society of Chemistry. (**c**) Schematic diagrams of annular Cu_3_Sb_0.88_Sn_0.10_Bi_0.02_Se_4_–based TE modules, reprinted with permission from ref. [153], copyright 2017 The Royal Society of Chemistry. (**d**) The Mini-PEM used to measure the conversion efficiency of a segmented Cu_12_Sb_4_S_13_–based single-leg, and (**e**) experimental power generation efficiency for the segmented leg. The insets are the fabricated TE single-leg. Reprinted with permission from ref. [103], copyright 2021 Wiley-VCH GmbH. (**f**) Experimental TE conversion efficiency *η* and (**g**) simulated *η* by COMSOL Multiphysics software for Cu_3_SbS_4_–based single-leg module, the inset is a photo of mini-PEM test. Reprinted with permission from ref. [28], copyright 2023 Wiley-VCH GmbH.

For practical TE applications, moving from high-performance materials to high-efficiency devices is of great significance. CBDL compounds conform to the concept of green environmental protection and have great practical application value while the absence of *n-*type conductive compounds greatly hinders the manufacture and application of CBDL-based TE devices. During the journey of device development, researchers have made a lot of efforts. In 2017, Qiu et al. [185] manufactured a CBDL-based TE module via integrating high-performance *n-*type Ag_0.9_Cd_0.1_InSe_2_ and *p-*type Cu_0.99_In_0.6_Ga_0.4_Te_2_ leg, respectively, as shown in Figure 14a. The output power of module reached 0.06 W under a temperature difference of 520 K (Figure 14b), demonstrating that diamond-like compounds are also potential candidates for TE applications. On the foundation of obtaining high-performance in (Sn, Bi)-codoped nanocrystalline Cu_3_SbSe_4_ materials, Liu et al. [153] fabricated a hot pipe integrated by a series of ring-shaped Cu_3_Sb_0.88_Sn_0.10_Bi_0.02_Se_4_–based TE modules (Figure 14c), which can be used for the purpose of retrieving the waste heat from exhaust gas pipes in vehicles. Moreover, Li et al. [103] synthetized a segmented Cu_12_Sb_4_S_13_–based single-leg module, which had a superior conversion efficiency *η* of 6% at Δ*T* = 419 K, as shown in Figure 14d,e. Recently, the Cu_3_SbS_4_–based single-leg module synthetized by Zhang et al. [28] approached a conversion efficiency *η* of 2% with Δ*T* = 375 K, which reached to 5.5% predicted by the COMSOL simulation analysis (Figure 14f,g). Apart from realizing excellent TE efficiency, good thermal stability is also crucial for the manufacturing of TE devices. In practice, the volatilization induced softening and decomposition is the core issue for thermoelectric selenides and sulfides working at elevated temperatures. In the latest research from Zhou’s group [163] demonstrated that the compositing of CuAlS_2_ significantly optimized the thermal stability of Cu_3_SbSe_4_–based compounds by pushing the decomposition temperature to a higher value, while also greatly improving the mechanical properties of the material. Eventually, a maximum *η* over 3% was achieved at a Δ*T* = of 367 K and an *I* = 0.8 A. Based on the above research, it seems that CBDL has considerable TE performance and has gradually attracted researchers’ attention in the field of practical TE applications.

## 6. Conclusions and Perspectives

By reviewing the research on copper-based diamond-like thermoelectric materials, it has been found that diverse compounds appear to have excellent TE performances as well as possessing *zT* higher than unity and an even approach to two. Advanced approaches to guide the development of new high-performance CBDL materials have been found, such as machine learning, high-throughput and union-*η* rules. There are also various approaches to improving the TE properties of CBDL compounds. It is worth considering that, during the process of optimizing electrical and thermal transport behaviors of TE materials, the regulation is never carried out separately, but that coordination and unification of the two are sought. Based on the efforts of researchers, the CBDL compounds have been greatly developed. There is no escaping the fact that the softening and decomposition of Cu-based compounds occurs when the compounds are exposed to high temperatures. Therefore, compared with practical materials, the CBDL compounds still have great room for improvement.

Considering practical applications, it is of great significance to shift our focus from high-performance TE materials to highly efficient devices. The integration for TE equipment requires both high-performance *n-*type and *p-*type legs. Currently, CBDL compounds are mostly *p-*type materials, while the further development of *n-*type CBDL compounds is beneficial for its TE application. In addition, in the research and development process of TE devices, it is also necessary to consider the comprehensive properties such as thermal stability, processability and self-compatibility. Therefore, the feasibility of manufacturing efficient TE devices based on CBDL materials remains a highly challenging issue.

The exploration of material properties is still ongoing, and the practical application of devices also needs to be developed. There has been a deep understanding of the transport mechanism of TE materials with the iteration and update of characterization methods, accompanied by the assistance of more advanced manufacturing technologies, and that the development of high-performance TE materials and devices based on CBDL compounds has a bright future.

## Data Availability

Not applicable.
